# Novel immersive virtual reality experiences do not produce retroactive memory benefits for unrelated material

**DOI:** 10.1177/17470218221082491

**Published:** 2022-03-10

**Authors:** Jörn Alexander Quent, Richard N Henson

**Affiliations:** 1 MRC Cognition and Brain Sciences Unit, University of Cambridge, Cambridge, UK; 2 Department of Psychiatry, University of Cambridge, Cambridge, UK

**Keywords:** Immersive virtual reality, novelty, behavioural tagging, memory, recollection, familiarity

## Abstract

The experience of novelty can enhance memory for information that occurs close in time, even if not directly related to the experience—a phenomenon called “behavioural tagging.” For example, an animal exposed to a novel spatial environment shows improved memory for other information presented previously. This has been linked to neurochemical modulations induced by novelty, which affect consolidation of memories for experiences that were encoded around the same time. Neurophysiological research in animals has shown that novelty benefits weakly encoded but not strongly encoded information. However, a benefit that is selective to weak memories seems difficult to reconcile with studies in humans that have reported that novelty improves recollection, but not familiarity. One possibility is that the novelty increases activity in hippocampus, which is also associated with processes that enable recollection. This is consistent with another prediction of behavioural tagging theory, namely that novelty only enhances consolidation of information that converges on the same neuronal population. However, no study has directly explored the relationship between encoding strength and retrieval quality (recollection versus familiarity). We examined the effects of exposure to a novel immersive virtual reality environment on memory for words presented immediately beforehand, under either deep or shallow encoding tasks, and by testing both recall memory immediately, and recognition memory with remember/know instructions the next day. However, Bayes factors showed no evidence to support the behavioural tagging predictions: that novelty would improve memory, particularly for shallowly encoded words, and this improvement would differentially affect familiarity versus recollection.

## Introduction

How does experiencing something novel influence our memory? It is well established in both human and non-human animals that novel stimuli are remembered better than familiar stimuli (e.g., Ranganath & Rainer, 2003; Tulving & Kroll, 1995; van Kesteren et al., 2012). This novelty advantage is believed to be related to increased neuromodulatory influences of acetylcholinergic and noradrenergic systems (Ranganath & Rainer, 2003) and of dopaminergic regions in the midbrain (Bunzeck & Düzel, 2006; Lisman et al., 2011). According to one framework (e.g., Lisman & Grace, 2005), novelty is detected in the hippocampus, which sends a signal to the ventral tegmental area, leading to release of dopamine in the hippocampus via dopaminergic back projections, which in turn lowers the threshold for learning (Schomaker, 2019).

Interestingly, novelty can not only enhance memory for the novel information itself, but also affect memory for other, unrelated information that occurs in temporal proximity to the novel information (Fernández & Morris, 2018). This enhancement of memory for information occurring either before or after the novel experience has been shown in both non-humans (Ballarini et al., 2009; Moncada & Viola, 2007) and humans (Ballarini et al., 2013; Fenker et al., 2008; Schomaker et al., 2014; though see also Biel & Bunzeck, 2019). Novelty-related memory enhancement has been found in a variety of paradigms: inhibitory avoidance (Moncada & Viola, 2007), spatial memory (Wang et al., 2010), spatial object recognition (Ballarini et al., 2009), contextual fear conditioning (Ballarini et al., 2009), conditioned taste aversion (Ballarini et al., 2009), story and picture recall (Ballarini et al., 2013), and word learning (Fenker et al., 2008; Schomaker et al., 2014). In possibly the most real-world application of this novelty effect in humans, Ballarini et al. (2013) showed that memory of primary school children was enhanced if they took part in special science or music lessons that were designed to be novel. The enhancing effect was observed for the learning of other, unrelated verbal and pictorial information, provided the novel lesson took place within an hour before or after such learning, consistent with a critical time window during which the novelty effect operates (see below).

One neurobiological explanation for the effect of novelty on surrounding information is provided by “behavioural tagging theory” (BTT; Ballarini et al., 2009; Moncada & Viola, 2007), which itself derives from the physiological mechanisms proposed by the synaptic “tag-and-capture” theory (Frey & Morris, 1997; Redondo & Morris, 2011). Briefly put, this theory postulates that two main steps are important to maintain late-long-term potentiation (late-LTP). First, a synapse is tagged because it has received input. In the second step, the tagged synapse needs to capture so-called plasticity-related products (PRPs) to induce the lasting structural changes that give rise to late-LTP. Experimentally, it can be shown that strong tetanisation of a synaptic input can produce both tagging and subsequent PRP capture. Weak tetanisation of a synaptic input, on the other hand, induces early-LTP, but this is not maintained unless the second stage of PRP capture occurs. One way to produce this PRP capture is to provide a second, strong tetanisation to a different synaptic input on the same population of neurons. In that case, both synaptic inputs benefit from the provision of PRPs and hence late-LTP is maintained. Something similar to strong tetanisation can potentially come from a different, but highly novel input, leading to a similar maintenance of late-LTP (Li et al., 2003; Straube et al., 2003; Straube et al., 2003). Both the induction of LTP in the hippocampus and behavioural tagging are dopamine dependent (Li et al., 2003; Wang et al., 2010), consistent with the aforementioned idea that dopamine is crucial for novelty-related memory enhancement (Lisman & Grace, 2005; Lisman et al., 2011).

Within the “tag-and-capture” theory, the lifetime of a tag is limited to approximately 90 minutes (Redondo & Morris, 2011), requiring the weakly learned information and strong tetanisation to co-occur within that time window. Likewise, behavioural tagging only occurs within a certain time window (Ballarini et al., 2009; Moncada & Viola, 2007). For instance, weak inhibitory avoidance training that normally only leads to spatial short-term memory (STM) can be strengthened to long-term memory (LTM) if animals are allowed to explore a novel, open field within up to an hour of that training (Moncada & Viola, 2007). However, the timescale of the behavioural tagging window depends on the task characteristics and may even have a nonlinear expression, given that some animal studies have shown that novelty that is too close to the unrelated encoding event does not enhance memory (Moncada et al., 2015). Information about the temporal dependencies in humans is scarce however, and several studies have shown memory enhancement when a novel experience occurs within a few seconds of the learning experience (e.g., Bunzeck & Düzel, 2006; Schomaker et al., 2014; though see Biel & Bunzeck, 2019).

In addition to the time between the encoding of critical information and the novel experience, a second consideration is the time between the novel experience and the subsequent test of memory (retention interval). Most animal models assume that a period of consolidation is required, such that the effects of novelty only emerge after a delay. However, in humans, Bunzeck and Düzel (2006) showed that presenting familiar images at the same time as novel images led to an overall increase in memory performance for the familiar images in a subsequent recognition memory task, but only when recognition was tested immediately; not when tested the next day (see also Biel & Bunzeck, 2019, for a failure to find an effect of novelty the next day). The effect of retention interval, and possible role of consolidation, therefore remains unclear. Here we tested both immediate recall and delayed recognition memory.

According to BTT, there are at least two further boundary conditions for behavioural tagging. First, as noted above, novelty does not enhance memory traces that are already strong, presumably because they already sufficiently captured PRPs (Moncada & Viola, 2007). This may explain why the effect of novel lessons on children in the above Ballarini et al. (2013) study was most pronounced for difficult information, which presumably would have only led to weak memories otherwise. It is also consistent with recent findings related to stress. Like novelty-related memory enhancement, stress-related memory enhancement has been linked to processes akin to those hypothesised in tag-and-capture theory (Bergado et al., 2011; McIntyre et al., 2012; Richter-Levin & Akirav, 2003). For example, spatial recognition memory in rats was promoted from STM to LTM by acute stress after weak but not after strong training (Lopes da Cunha et al., 2019), and stress-related increases of cortisol in humans only predicted memory for weakly learned neutral words, but not for strongly learned reward-predicting words (Quent et al., 2018; see Dunsmoor et al., 2015, for similar effects using Pavlovian fear conditioning). Here, we tested this directly by manipulating the encoding of words during the initial study phase, by using deep encoding task on one half of the words and a shallow encoding task on the other (Craik & Lockhart, 1972). If BTT is correct, the effect of novelty should be larger for shallowly than deeply encoded words.

However, the suggestion that novelty preferentially aids weak memories is less easy to reconcile with other human studies. For example, Fenker et al. (2008) used a paradigm similar to Bunzeck and Düzel (2006) to demonstrate that presenting novel images enhanced memory for unrelated words, but they only found this enhancement for words whose recognition was accompanied by a “Remember” judgement; the advantage was not seen for words whose recognition was accompanied by a “Know” judgement. Remember judgements are given to items for which some episodic aspect of their prior presentation is recalled (e.g., the spatiotemporal context or internal thoughts at the time); Know judgements are given to items that seem familiar, but their episodic context is not recalled (Tulving, 1985). Remember and Know judgements are associated with the theoretical concepts of recollection and familiarity, and while these concepts are not synonymous with memory strength (Yonelinas, 2002), few would contest that items judged familiar have, on average, weaker memory representations than those recollected. It is possible that some words in Fenker et al.’s study were initially encoded weakly, but the novel experience boosted them sufficiently that they were later recollected. However, it also seems likely that some words could have been encoded so weakly that they would not be recognised at all (i.e., missed), had a novel experience not boosted them such that they at least seemed familiar. In this case, an effect of novelty would be expected on Know judgements as well as Remember judgements, and possibly more so, if Know judgements are a better indicator of items that were initially encoded weakly.

One possible explanation for these results relates to a second boundary condition of BTT: that information must converge on the same neural population that is activated by the novel experience, in order for that information to benefit from the novelty-induced PRPs (Ballarini et al., 2009). For instance, open field exploration does not enhance conditioned taste aversion in rats, but a novel taste does (Ballarini et al., 2009). Here it is important to distinguish conceptually unrelated (e.g., word learning and spatial navigation) and neuronally unrelated. In its most basic form, BTT postulates that detection of novelty leads to a dopaminergic signal that boosts encoding in places that receive that signal. It is therefore conceivable that novelty is detected by one neural population within a brain region (e.g., the hippocampus), but a wide-spread signal is also received by other populations within that region, including the population that is encoding task-relevant information. Therefore, information that is conceptually unrelated to the novel experience can still benefit, providing the information is neuronally related in the sense of receiving the same memory-boosting signal. Given the above evidence that hippocampus is important for detecting novelty, and other evidence that the hippocampus is important for encoding the spatiotemporal and associative context that defines recollected memories, then it is possible that a novel experience only improves recollection of information (as in Fenker et al., 2008). We therefore included Remember/Know judgements in a test of recognition memory, to investigate whether novelty has selective effects on one or the other type of memory.

A further consideration is the nature of the novel experience. Previous human studies have used novel images or films, and at least one of these (Biel & Bunzeck, 2019) recently failed to find an effect of novelty. This study compared the effects of watching novel versus familiar films, and the authors speculated that the lack of difference was because the films did not engender active engagement, at least to the level engendered by the exploration of a novel spatial environment used in many animal studies. Furthermore, if the hippocampus is key for the novelty effect, active navigation might be important for maximally engaging the hippocampus, given its role in navigation (O’Keefe & Nadel, 1978). One way to expose humans to a novel environment, but within the controlled setting of a laboratory, is to use virtual reality (VR). Indeed, another human study used VR to compare novel versus familiar environments in their effects on words learned immediately after the VR experience (Schomaker et al., 2014). Exploring a novel environment, relative to a familiar one, enhanced free recall of the words, though not recognition memory for the words (though these authors did not distinguish recollection versus familiarity in their recognition task). Since recall relies more heavily on recollection, these findings are consistent with Fenker et al. (2008), on the assumption that their recognition performance was dominated by familiarity.

Given the importance of these findings for education and other real-world situations, we attempted to replicate the effects of a novel spatial navigation experience on memory for unrelated words, as a function of the encoding task (deep vs. shallow) and retrieval quality (recognition with remember vs. know judgements, plus recall). One half of the words were encoded deeply using a animate/inanimate task (like Fenker et al., 2008), while the other half were encoded shallowly using an alphabetical task, which results in worse memory, i.e., weaker encoding (Craik & Lockhart, 1972; Otten et al., 2001; Yonelinas, 2002). Encoding was incidental, i.e., we did not tell participants that their memory for the words would be tested later. Like Schomaker et al. (2014), we also used VR, but in particular an immersive VR system in which participants can physically walk around a virtual room (rather than navigating with a mouse and keyboard, as in Schomaker et al., 2014). Due to the current rarity of immersive VR systems (compared with 2D games or even passive VR), we expect this to be a highly novel experience (and we excluded people who have experienced immersive VR before). Indeed, in our prior work with immersive VR, most participants were amazed by their experience. Immersive VR also renders the novel experience more similar to the open field exploration used to induce novelty in non-human animals. To isolate the novelty of the experience from the sensory, motor, and executive demands of the VR task, the control group had experienced the same VR task the day before, so it was no longer novel. In summary, according to BTT, we expect to find (1) a basic novelty effect (better memory for the preceding words in the novel group vs. control group), (2) a greater novelty effect for shallowly than deeply encoded words, and (3) a novelty effect that is either larger or smaller for recollected words (Remember judgements) relative to words judged as familiar (Know judgements).

## Methods

### Participants

All participants were recruited from MRC Cognition and Brain Sciences’ SONA system, in-house participant panel, or through word-to-mouth. This study was approved by the Cambridge Psychology Research Ethics committee (PRE.2018.107). Data collection was stopped after data from 72 participants led to BF_10_ > 6 or < 1/6 for one of our planned comparisons (see Results), as registered. Participants were paid £6/h, and they received up to £3 for travel compensation per visit. Full payment was only made after successful completion of Day 3. Note that data collection for this project had to be paused due to the COVID-19 pandemic. Approximately half of the participants completed the task before the pandemic and the rest after. The group ratio (novelty vs. control) at the beginning of the pandemic was circa 2.2 to 1.

Eighty-two participants were tested in total. Ten were excluded for the following reasons: three participants did not complete the recognition task the next day, two had technical failures, two data sets were invalid due to experimenter error, one felt unwell after Day 1, one had prior VR experience (a registered exclusion criterion), and one participant’s Pr was at chance level, as defined by the bootstrapping procedure. A further two participants were included, except for tests for which they had missing data: one had missing recall data and the other missed the VR questionnaire. Furthermore, two participants did not use the correct keys so their data had to be excluded from the analysis of the encoding task.

The final sample size of 72 participants (36 per group; see power analysis below) contained 53 females, 18 males, 1 non-binary, with mean age *M* = 26.3 years (*SD* = 6.25 years). Participants in the Novelty group completed the online recognition task *M* = 25.5 hours (*SD* = 2.38 hours) after encoding, while the Control group completed the task after *M* = 25.1 hours (*SD* = 2.17 hours).

### Procedure

**Figure 1.**
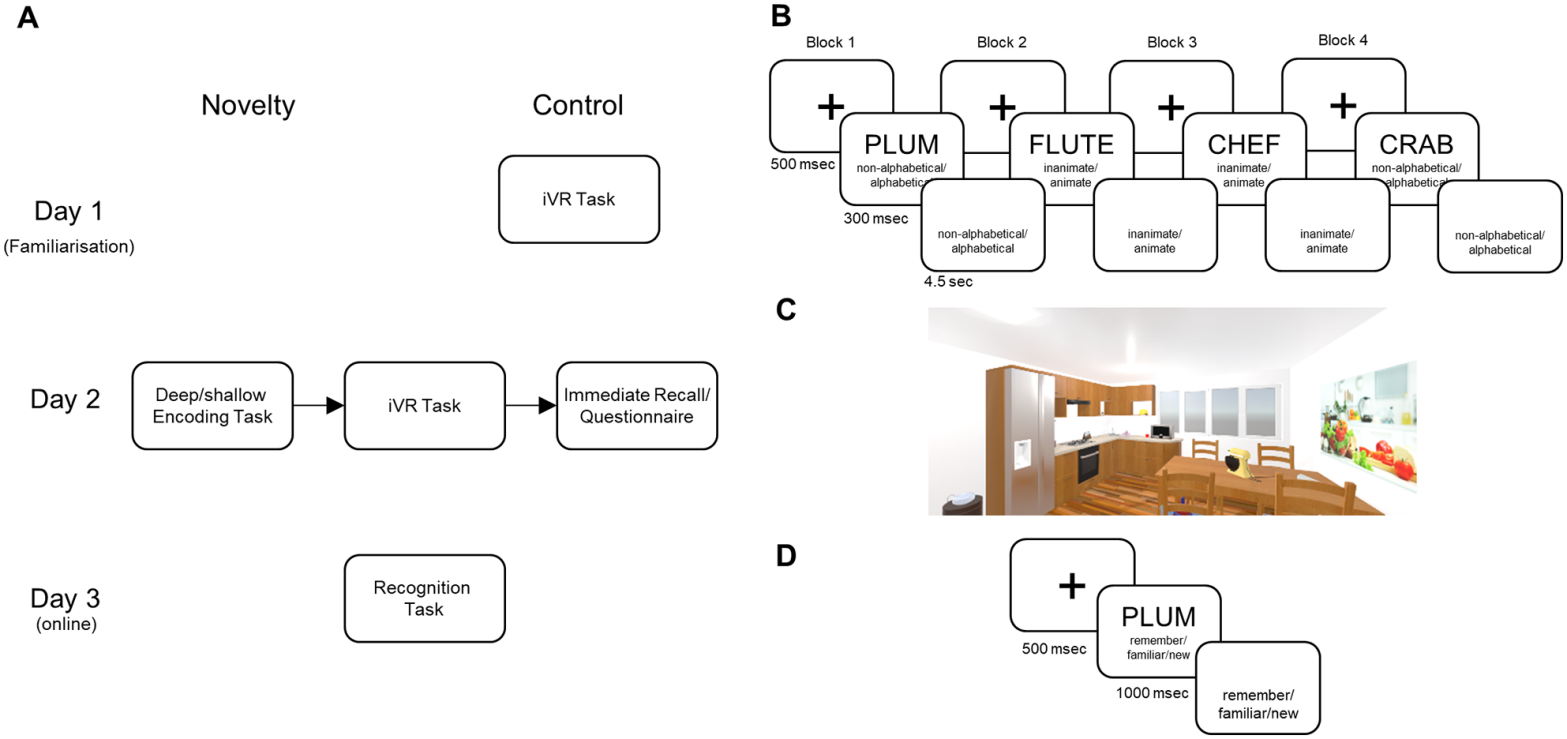
(A) Illustration of experimental design. On Day 1, the control group was familiarised with the immersive virtual reality (iVR) task (B). Day 2 is the same for both the novelty and the control group. It started with the deep/shallow encoding task (C), immediately followed by the iVR task. This task was novel to the novelty group but familiar to the control group. The iVR task was followed by immediate recall for 5 min and the “Igroup” presence questionnaire (Schubert et al., 2001). On Day 3, participants were asked to complete the memory task online during which they completed a remember/know/new recognition task (see D). (B) Illustration of encoding task. A trial of the encoding task started with the presentation of a fixation cross (500 ms) followed by the presentation of word (300 ms) along with reminder of current task: either deep (inanimate/animate) or (non-alphabetical/alphabetical). Participants had 4.5 s to respond. This task had four blocks following an ABBA design, with A/B referring to task. The task and stimuli were taken from Otten et al. (2001). (C) Screenshot of virtual kitchen of the iVR task with objects present as seen by the participants. (D) Illustration of recognition memory. A trial of recognition task started with a fixation cross (500 ms) followed by the presentation of the word (1000 ms) alongside a task reminder (remember/familiar/new) that stayed on screen until a response of given.

In terms of stimuli (see below) and procedure, we closely followed a previous paper by Otten et al. (2001), ensuring that memory performance was in the correct ballpark. The experiment ran over 3 days (see Figure 1), with Days 1–2 in the laboratory and Day 3 at home. On the critical Day 2, the experimental procedure can be divided into three phases: study, immersive virtual reality (iVR), and test phase. In the study phase, participants incidentally encoded words. The words were presented in four blocks, with one of the two study tasks (see below) in each block (counterbalanced across participants as ABBA or BAAB). Then they performed the iVR task (details below). The only difference between the Novelty and the Control group is that the Control group had already completed the iVR task the day before. Finally, they freely recalled as many of the words as possible and completed a short questionnaire about the iVR experience. On the final Day 3, participants performed a recognition task with remember/know/new judgements to distinguish studied versus new (unstudied) words. We decided to test recall only on Day 2 and recognition memory only on Day 3 to minimise retrieval-induced enhancement or forgetting (Anderson et al., 1994).

During the study phase, a trial started with a fixation cross displayed for 500 ms followed by the presentation of a word for 300 ms alongside a reminder of the current task (alphabetical vs. animate). The tasks were based on Otten et al. (2001): In the alphabetical (shallow) tasks, participants decided whether or not the first and the last letter of a word are in alphabetical order, while in the animate (deep) task, they decided whether or not the presented word refers to an animate object. Participants were instructed to use one finger from each hand to press one key for non-alphabetical/inanimate words and another for alphabetical/animate words. The reminder remained on the screen for 4.5 s, comparable to Otten et al., after which the next trial started. A failure to respond in that time frame led the trial being scored as no response. The encoding task was divided into four blocks of 72 words, with each block having one of the two tasks. The order of words within a block was randomised once and then was the same for every participant. Participants were not told that their memory for the words will be tested later; rather, they were told that their ratings of the words would simply help prepare the stimuli for another experiment on different participants.

After the study phase, participants of both groups (Novelty and Control group) immediately spent approximately 25 min in an iVR task. Assignment of participants to groups alternated according to availability. The iVR task consisted of an encoding phase, in which participants have 45 s to explore a virtual kitchen and memorise the locations of 20 everyday items (e.g., microwave, helmet etc.; see Figure 1B). After this, participants were taught how to pick up objects in this virtual environment (VE) and then asked to pick up and place each of the 20 objects that they had seen earlier at the locations that they had to remember. After completing this, participants removed the VR headset and completed a 3 Alternatives Free Choice (3AFC) recognition memory task, in which participants had to choose the correct object location out of three alternatives, and a rating task, in which participants rated how expected the locations and objects were. More information on the nature of the iVR task can be found in Open Science Framework pre-registration form: https://osf.io/4sw2t/. The visuospatial nature of the iVR task (i.e., remembering object locations) was sufficiently different from memorising single words that we did not expect direct interference between the tasks (Wixted, 2004).

After the iVR task, participants were asked to recall as many words as they can (immediate recall) for 5 min. Participants were asked to write down the words that they remember on a piece of paper. After this, participants completed the Igroup Presence Questionnaire (IPQ; Schubert et al., 2001) to assess involvement, presence, and realism of the iVR experience (http://www.igroup.org/pq/ipq/items.php). In a computerised adaptation of this questionnaire, participants gave their ratings on a slider scale using the original questions and anchors.

The next day, participants were asked to complete a recognition memory task similar to the one used by Fenker et al. (2008), in which the participants were presented with the words they studied previously, intermixed randomly with 144 non-studied words. A trial in this task started with a fixation cross displayed for 500 ms followed by the presentation of the word for 1,000 ms. Participants were instructed to press the “n” key if they think the word was new, the “f” key if the word was familiar, and the “r” key if they remembered the word. Note that we preferred to use the word “familiar” when instructing participants because they typically find it easier to understand than the word “know.” The exact instructions were based on those used by Bastin et al. (2010), though conceptually our results were comparable to previous studies using Tulving’s original “know” instruction, which is why we continued to use the words Remember and Know in analyses below. At the end of the online session, participants were completely debriefed and compensated.

### Stimuli

The same number of words (144 per task, i.e., 288 total) were used in the study phase as in Otten et al. (2001), though only half of the number of unstudied words (144) were used in the test phase, since unstudied words were not of primary interest here. Thus, a subset of 432 words from Otten et al. (2001), balanced according to study response category (animate/inanimate and alphabetical/non-alphabetical), had been split into three sets of 144 words and the assignment of words to the two study conditions and the unstudied condition were counterbalanced across participants (six different combinations in total). The lists had been selected so as not to differ in terms of the characteristics available in the MRC Psycholinguistic Database (see here for selection and list creation process; for words see Wilson, 1988).

For the iVR task, the following stimuli will be used: the virtual kitchen has been created using SketchUp (https://www.sketchup.com/), unity3d (https://unity3d.com/), and freely available 3D models downloaded from https://archive3d.net. In addition to typical kitchen furniture such chairs and a table, this kitchen contains 20 everyday objects such a hat, a calendar, and a toy (for an illustration of the VE, see Figure 1B).

### Equipment

At the beginning, the VE was presented with an HTC VIVE VR system and run on MSI VR ONE 7RE-057UK computer with Intel Core i7-7820HK, 16 GB RAM, and GeForce GTX 1070, which can be worn as a backpack allowing free movement. Due to equipment failure we had to replace the VR computer and started to use a Dell Desktop PC (Precision 5820 Tower X-Series) with Intel Core i9-10900 and GeForce RTX 2080 midway through data collection. To allow free movement, we then used the VIVE Wireless Adapter.

Other laboratory tasks were completed on a Dell Latitude E6530 laptop. For these tasks, stimuli were presented with Matlab (https://www.mathworks.com) using the Psychophysics Toolbox extensions (Brainard, 1997; Kleiner et al., 2007; Pelli, 1997). The online tasks were run on a JATOS (Lange et al., 2015) server hosted on the MRC-CBU servers, which were compliant with data protection and security policies. The task was programmed in Javascript with jsPsych (de Leeuw, 2015).

### Statistical design and hypotheses

To address the hypotheses outlined in the Introduction, we ran Bayesian *t* tests (Morey & Rouder, 2018) using the package *BayesFactor* (version 0.9.12-4.2) for both recall and recognition tasks, with factors novelty (Novelty vs. Control group, between-participants), encoding task (shallow vs. deep, within-participant) and, for the recognition task, memory quality (probability of recollection vs. familiarity, within-participants). The hypotheses were tested using Bayes factors (BFs), for the alternative versus the point-null hypothesis, calculated for *t* tests using the default scale parameter of √2/2. We used between-participant *t* tests to test for the presence of main effects and interactions, given our directional predictions (note that, in factorial designs, within-participant factors with only two levels can be reduced to difference scores, enabling all interactions in the present design to be reduced to *t* tests between the two groups on these difference scores; likewise, main effects can be reduced to *t* tests between the two levels of one factor by aggregating across the other factors). Using *t* tests for planned comparisons, instead of the more traditional ANOVA approach, enables us to accurately express our statistical hypotheses that are directed in some cases. Specifically, we predicted a main effect of novelty in recall (Hypothesis 1.1) and recognition memory (Hypothesis 1.2), with better memory in the novelty group; an interaction between novelty and encoding task in recall (Hypothesis 2.1) and recognition (Hypothesis 2.2), with a larger novelty effect predicted for words that are shallowly encoded; an interaction between novelty and memory quality in recognition memory (Hypothesis 3), with different probabilities of recollection and familiarity in the Novelty versus Control group; and the three-way interaction between novelty, encoding task, and memory quality in recognition memory (Hypothesis 4), with different probabilities of recollection and familiarity restricted to the shallowly encoded words in the Novelty group. Note that the first two planned comparisons are one-tailed, while the last two are two-tailed. The directional predictions are explained in the Introduction, and we argue that directional hypotheses were justified because, according to BTT, null effects would be equivalent to negative effects and would lead to the same conclusions. In other words, that novelty could impair memory is not interesting theoretically to us other than that it will provide evidence against the BTT. This is not necessarily true for the two last comparisons because, as explained in the Introduction, different boundary conditions of BTT predict that novelty either boosts familiarity or boosts recollection.

**Figure 2.**
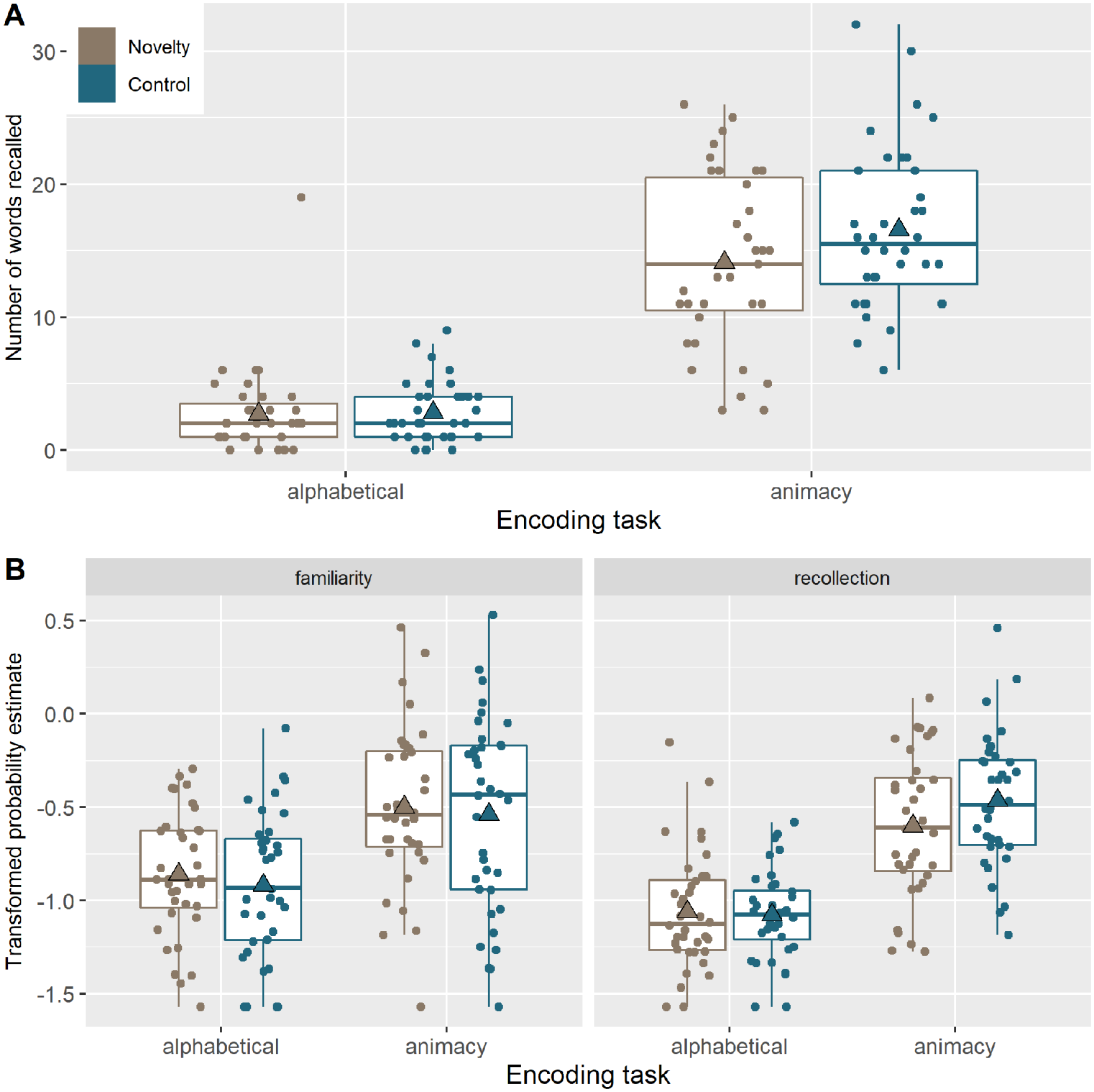
Box plots for recall and recognition. (A) Number of words recalled as a function of the encoding task (alphabetical/non-alphabetical and animate/inanimate) for the novelty group (brown) and the control group (blue). (B) Arcsine transformed probability estimates from the Multinomial Processing Tree (MPT) analysis of the recognition task. Estimates for familiarity (left) and recollection (right) are displayed as function of encoding task for the novelty and the control group. The horizontal line represents the median, the triangles represent the mean, the boxes represent the 25th and 75th percentile, while the whiskers show the default 1.5 × interquartile range from the hinge.

For the recall data, the dependent variable was the number of studied words recalled. For the recognition memory data, we used a multinomial processing tree (MPT) model that is analogous to the “Source-Item” model in Cooper et al. (2017), which assumes two underlying processes contributing to memory: the probability of recollection (*r*) and the probability of familiarity (*f*) (see Figure 2). In this model, recollection and familiarity are discrete states and recollection is always accompanied by familiarity. Additional parameters that are estimated but not subject to statistical test in our model are *gr* and *gk*, which are the probabilities that a guessing response leads to a Remember and Know response, respectively. Estimating these parameters effectively adjusts the estimates of recollection and familiarity by their false alarm rates. The MPT will be fit using the *MPTinR* package (version 1.11.0; Singmann & Kellen, 2013). For statistical analysis of the resulting parameters, probabilities were submitted to an arcsine transformation so that their values approximately follow a normal distribution and are not bounded between 0 and 1. Note that, while the statistical tests and effect size estimates for all proportions/probabilities are based on arcsine transformed values, raw accuracy rates and probability estimates are reported in the text. Note also that we neglected to mention in our registration any basic tests that do not distinguish between recollection and familiarity, e.g., that our encoding task manipulation had its intended effect on memory. For these, we used Pr (hit − false alarm rate), which is equivalent to what would be obtained from an MPT with no remember/know branches.

In terms of choosing participant numbers, we ran a “fixed N” Bayesian analysis (Schönbrodt & Wagenmakers, 2018) for the hypotheses above. We based our sample size estimation on simulating a general linear model (GLM) with effect sizes similar to those reported in previous studies for a main effect of novelty. Based on the reported statistics, we calculated Cohen’s *d* for four effects that are in the literature. For Fenker et al. (2008), the effect size is *d* = 0.588 for immediate remember, *d* = 0.943 immediate recall, and *d* = 0.894 for delayed remember. For Schomaker et al. (2014), the effect size for immediate recall is *d* = 0.873. Our simulations based on the median (*d* = 0.884) showed that 36 participants per group is sufficient to provide compelling evidence (BF_10_ > 6) for one-tailed comparisons (Hypothesis 1 and 2) with a probability of approximately 91%, and for two-tailed comparisons (Hypothesis 3 and 4) with a probability of approximately 83%. At the same time, we would be able to provide compelling evidence (BF_10_ < 1/6) for the null hypothesis for the absence of an effect in 30% of the cases for one-tailed comparisons, while the probability of obtaining compelling misleading evidence is extremely low for all comparisons ranging between 0% and 0.09% (see https://github.com/JAQuent/noveltyVR/blob/master/preparation/powerAnalysis.md for whole design analysis).

## Results

### Encoding task

Before moving to the registered analysis of the memory data, we report an exploratory analysis of the encoding task. There was no compelling evidence that accuracy in the animacy condition, *M* = 0.884 (*SD* = 0.0864), was different from accuracy in the alphabetical condition, *M* = 0.897 (*SD* = 0.101), BF_01_ = 2.51, *d* = 0.183.

However, as expected by design (Otten et al., 2001), responses were faster in the animacy condition, *M* = 1000 ms (*SD* = 227 ms), compared with the alphabetical condition, *M* = 1540 ms (*SD* = 351 ms), BF_10_ = 2.51e + 21, *d* = 1.88. Therefore, any better retrieval in the animacy condition is unlikely to simply reflect a longer time spent studying.

### Memory tasks

**Figure 3.**
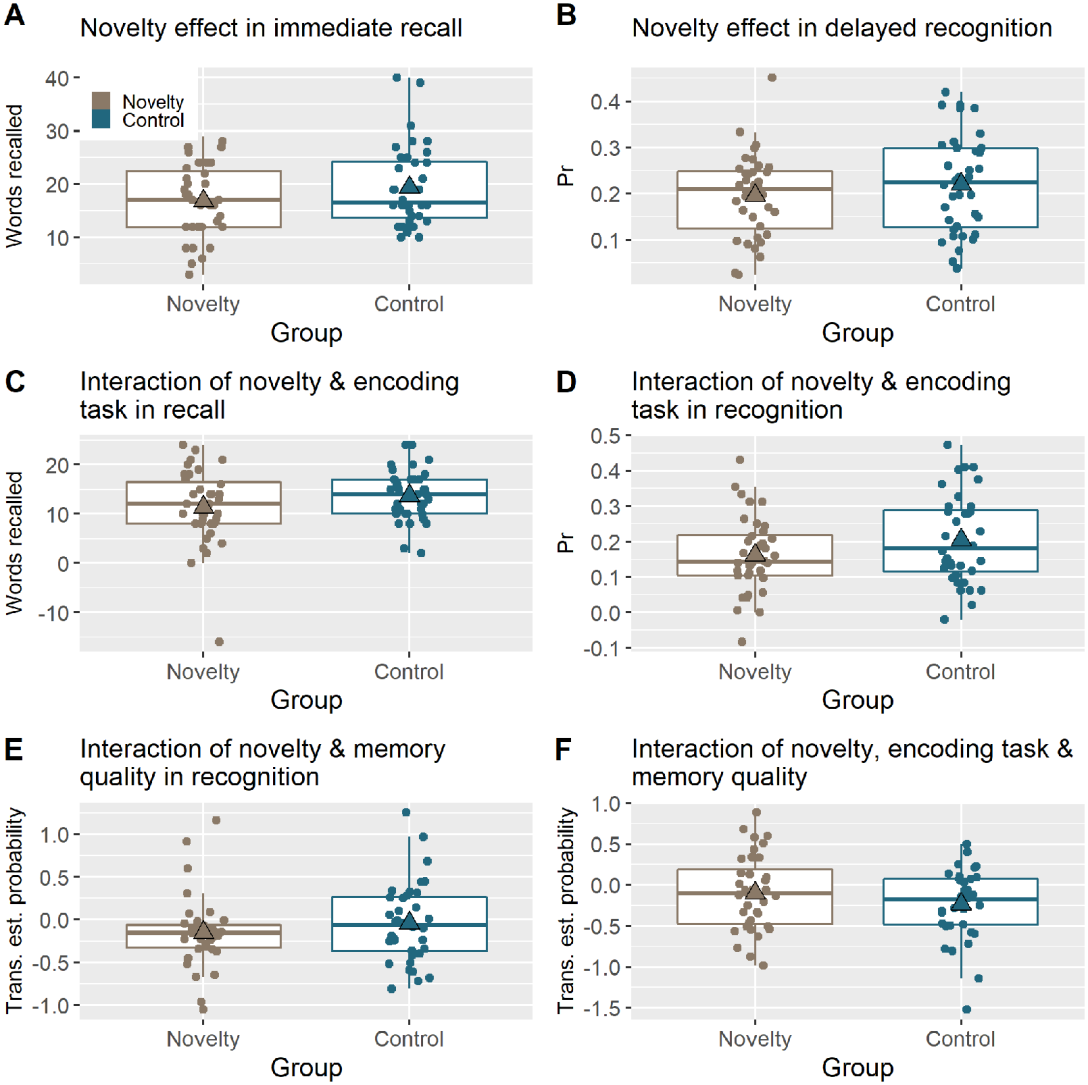
Boxplots for each hypothesis showing the values or difference scores that are compared with the Bayesian *t* tests. The novelty group is displayed in brown and the control group in blue. The y-axes for Panel E and F are short for transformed estimated probability.

Despite not explicitly registering in Stage 1, we first report the “levels of processing” effect to confirm that this manipulation had the desired effect of producing greater memory for deeply than shallowly encoded words. For the immediate recall, participants recalled more words in the animacy condition, *M* = 15.4 (*SD* = 6.38), than in the alphabetical condition, *M* = 2.77 (*SD* = 2.85), BF_10_ = 1.28e + 22, *d* = 1.91. Similarly for the delayed recognition test, memory performance (measured as Pr) in the animacy condition, *M* = 0.300 (*SD* = 0.145), was better than in the alphabetical condition, *M* = 0.116 (*SD* = 0.0747), $BF_{10}$ = 5.62e + 17, *d* = 1.56.Thus, our level of processing manipulation had the intended effect (though it is worth noting that the “levels of processing” effect might be exaggerated when manipulated within-participant, as here, owing to participants prioritising retrieval for the deep task over the shallow task, even when those tasks are blocked and memory encoding was incidental). Full results for recall and recognition task can be found in Figure 3.

For registered Hypothesis 1.1 (Figure 3A), the immediate recall tests provided evidence that participants in the Novelty group, *M* = 16.8 (*SD* = 7.02), did not recall more words than participants in the Control group, *M* = 19.4 (*SD* = 7.59), i.e., compelling evidence for the null hypothesis, BF_01_ = 9.2, *d* = –0.35. Based on this, we stopped data collection. In a two-tailed version of this test (not registered), evidence that the Control group actually recalled more words than the Novelty group was inconclusive, BF_10_ = 0.618, despite the difference in means.

For Hypothesis 1.2 (Figure 3B), an overall measure of Pr for the delayed recognition memory test provided compelling evidence that the Novelty group, *M* = 0.196 (*SD* = 0.091), did not have better memory than the Control group, *M* = 0.220 (*SD* = 0.106), BF_01_ = 7.72, *d* = –0.25.

**Figure 4.**
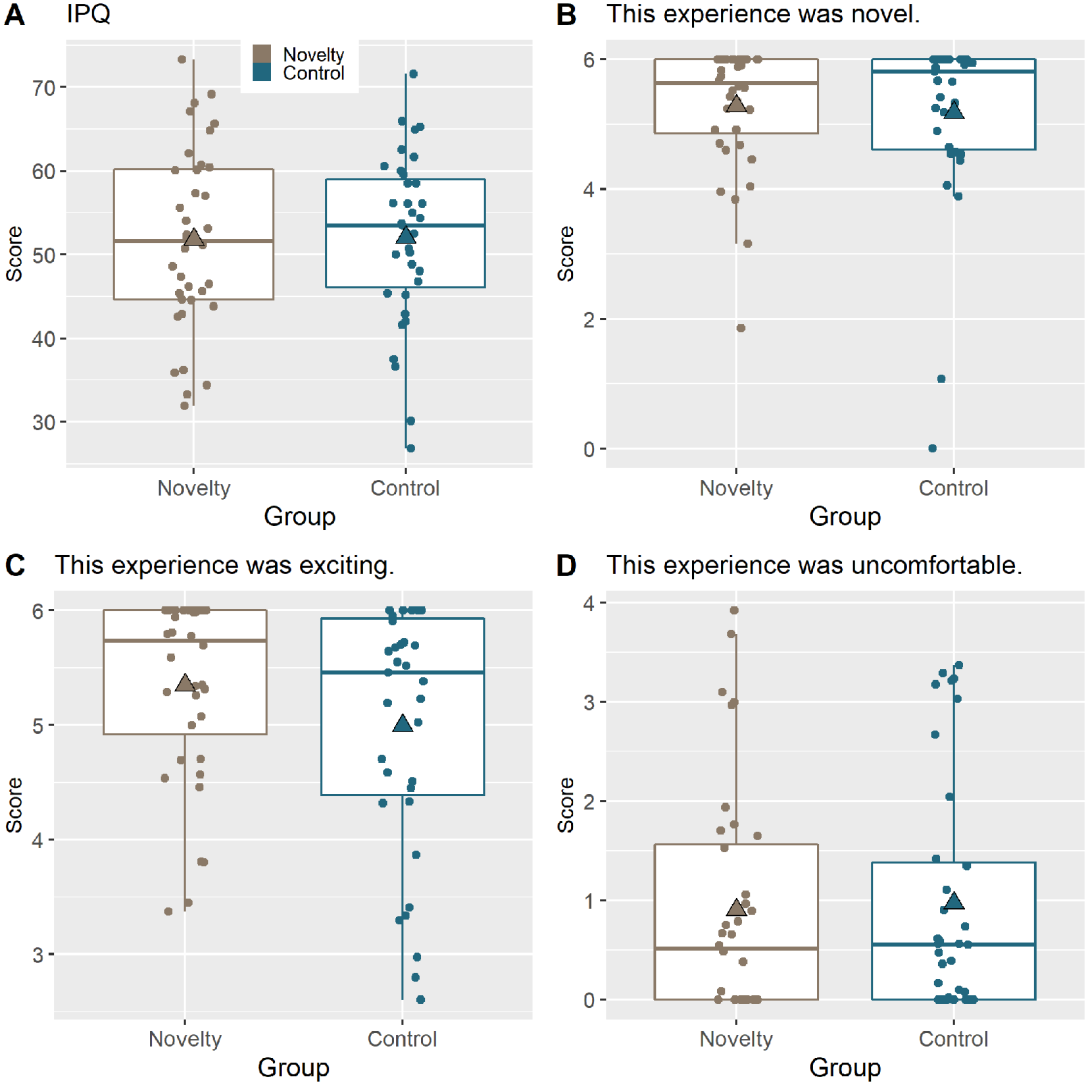
Boxplots for questionnaire data with triangle showing mean. The novelty group is displayed in brown and the control group in blue.

For Hypothesis 2, we found inconclusive evidence that the levels of processing effect for immediate recall differed between the Novelty group, *M* = 11.4 (*SD* = 7.71), and Control group, *M* = 13.7 (*SD* = 5.1), BF_10_ = 1.17, *d* = 0.356 (Hypothesis 2.1; Figure 4C), or that it differed for delayed recognition between the Novelty group, *M* = 0.162 (*SD* = 0.108), and the Control group, *M* = 0.205 (*SD* = 0.125), BF_10_ = 1.29, *d* = 0.371 (Hypothesis 2.2; Figure 3D).

For Hypothesis 3 (Figure 3E), we found inconclusive evidence (two-tailed) of a difference in recollection versus familiarity during delayed recognition (i.e., difference in the “*r*” and “*f*” parameters from the MPT, collapsed across two encoding conditions) between the Novelty group, *M* = –0.0509 (*SD* = 0.152), and Control group, *M* = –0.024 (*SD* = 0.164), BF_10_ = 0.385, *d* = 0.244.

To test for the interaction of Hypothesis 4 (Figure 3F), we first calculated the difference between the two encoding conditions (deep versus shallow) for each MPT parameter (*r* vs. *f*), then subtracted these difference scores and compared them across groups. Again, there was inconclusive evidence for any difference between the Novelty group, *M* = –0.0162 (*SD* = 0.195), and the Control group, *M* = –0.0715 (*SD* = 0.186), $BF_{10}$ = 0.486, *d* = 0.3. Table 1 shows a summary of BF for each hypothesis.

**Table 1.** A summary of the results of the six registered hypotheses.

Hypothesis	Description	BF_10_	BF_01_
1.1	Main effect of novelty in immediate recall	0.109	9.200
1.2	Main effect of novelty in delayed recognition	0.130	7.720
2.1	Interaction between novelty and encoding task in recall	1.170	0.855
2.2	Interaction between novelty and encoding task in recognition	1.290	0.774
3	Interaction between novelty and memory quality in recognition	0.385	2.600
4	Three-way interaction between novelty, encoding task and memory quality in recognition	0.486	2.060

### Post VR questionnaire

As an additional non-registered exploratory analysis, we examined the data from the post VR questionnaire. For this, the data were rescaled to vary from 0 to 6, as in the original scale (Schubert et al., 2001), with inverse items reversed (http://www.igroup.org/pq/ipq/data.php). The IPQ score was then calculated by summing across all items (similar to Schomaker et al., 2014, as confirmed by personal communication). This analysis showed that the Novelty group, *M* = 51.8 (*SD* = 10.9), did not differ from the Control group, *M* = 52 (*SD* = 10.1), *d* = 0.0238, BF_10_ = 0.246 (Figure 4A).

In addition, we asked participants to rate the statements: “This experience was novel,” “This experience was exciting” and “This experience was uncomfortable.” However, group differences did not arise for any of these statements: most surprisingly for Statement 1, concerning subjective rating of novelty, there was anecdotal evidence against the Novelty group, *M* = 5.28 (*SD* = 0.957), finding the iVR experience more novel than the Control group, *M* = 5.18 (*SD* = 1.34), *d* = 0.049, BF_10_ = 0.249, BF_01_ = 4.016 (Figure 4B). Note to deal with the negative skew for Statement 1, data were scaled from 0 to 1 and then transformed with arcsine transformation for the analysis only.

## Discussion

This study found evidence against the hypothesis that a novel experience can retroactively enhance memory for material that had been learned prior to that experience. For the novel experience, we used people’s first experience of an iVR environment (compared with other people’s second experience); for the material to be remembered, we used a list of unrelated words, which were studied under incidental tasks of either animate/inanimate judgement (deep encoding) or alphabetical judgement (shallow encoding). BFs showed compelling evidence that memory was not enhanced for same-day recall of the words (immediately after the iVR experience), and compelling evidence for no effect on recognition memory tested the next day. At the same time, we found no conclusive evidence (either way) for our additional hypotheses that any novelty-related boost would be greater for shallowly than deeply encoded words, and would differ for words later recollected versus familiar in the delayed recognition test.

The lack of any effects is surprising because previous studies did find that experiencing a novel VE versus a familiar one can boost memory for words learned after the VE experience (Schomaker et al., 2014; Schomaker & Wittmann, 2021) using a design very similar to ours. One reason could be that this type of novelty only has proactive effects on memory for this type of material, as Schomaker et al. found, but no retroactive effects, as here. However, this is contrary to animal studies that tend to find both proactive and retroactive effects, and contrary to the BTT derived from these animal data.

Despite the Schomaker et al. results, one might wonder whether novelty effects on memory are only seen when the novel material is comparable to the material to be remembered, i.e., within-modality, so would not be expected to extend from a novel iVR experience to memory for words. Indeed, BTT only predicts a memory benefit for material that is processed in the same brain region in which a novel experience has triggered plasticity-related proteins (PRPs). However, the argument made in our Introduction was that the spatial novelty of navigating in a VR environment (analogous to the novel arenas used to demonstrate the effect in rodents) triggers PRPs in the hippocampus; a brain region long-known to support navigation (O’Keefe & Nadel, 1978). However, the hippocampus is also well established as important for encoding the spatiotemporal context in which stimuli are encountered, regardless of the nature of those stimuli (e.g., Argyropoulos et al., 2021). Indeed, this was the basis of our secondary hypothesis that the novel iVR experience will selectively enhance recollection of words (i.e., retrieval of their context); and not affect simple familiarity of the words. Most importantly, a BTT explanation based on a brain region like the hippocampus being involved in both novelty and general memory encoding is necessary to explain previous cases of “cross-modality” novelty effects on memory (e.g., Ballarini et al., 2013; Fenker et al., 2008).

One reason why we did not observe a novelty effect could be because overall memory performance was too low, i.e., a floor effect in which there was insufficient range to detect an effect of novelty. Free recall was only around 5% of studied words, and recognition performance (in terms of Pr) was around 0.2, which is quite low. Nonetheless, we were able to obtain overwhelming evidence (BFs > 100) for our “levels of processing” manipulation (i.e., greater memory for deeply than shallowly encoded words) in both recognition and recall, suggesting that any range effects did not prohibit detecting some effects.

Another possibility is that our comparison of first versus second experience of iVR did not differ sufficiently in terms of novelty, either because the first experience was not sufficiently novel, or because the second experience was equally novel, i.e., the novelty of VR did not decline sufficiently in the Control group. This might explain why there was no evidence for a difference between our two groups in their mean rating of novelty (or any other aspect of their experience) in our post-experiment questionnaire, though the lack of difference in subjective ratings of novelty could be obscured by a ceiling effect. We chose this comparison of first versus second experience of a VE because it was tightly controlled, compared for example to contrasting an iVR experience to a more familiar, non-iVR experience, which could differ in ways other than novelty, and because a similar comparison was used by Schomaker et al. (2014; Schomaker & Wittmann, 2021). In fact, Schomaker et al. compared a novel VE with a familiar VE, for participants who could have been generally familiar with the technology, which if anything would seem a less extreme contrast in novelty than our comparison of participants’ first ever experience of iVR: i.e., our groups differed in their familiarity for both the VE (our virtual kitchen) and the iVR technology (since we excluded participants with previous iVR experience). All in all, the claim that our comparison was not novel enough seems to conflict with previous human studies that have simply used familiar versus novel static images on a computer screen (e.g., Fenker et al., 2008).

The lack of a difference between groups in the score given on our post-experiment novelty question could also have other reasons. Foremost, participants in the two groups may have used different references for their novelty rating (despite experiencing different levels of absolute novelty), e.g., participants in the Control group might have rated the novelty of their second iVR experience relative to other experiences that day (Day 2), rather than explicitly refer back to their first iVR experience on the previous day (Day 1); or they may have misunderstood the question, and rated their novelty for the overall experiment over the 2 days. It is also worth noting that most previous studies did not report subjective experiences of novelty for their manipulations. Schomaker et al. (2014) used the IPQ (Schubert et al., 2001), which is commonly used to measure presence, involvement, and realism in VR experiments. While presence ratings were higher in Schomaker et al. (2014) after being in a novel versus a familiar VE, this was not found in their subsequent study (Schomaker & Wittmann, 2021), and it is unclear how IPQ data relate to novelty per se.

Another possible reason for the lack of a novelty-boost on memory is that the boost was masked by the fact that there were differences between the Novelty and the Control group in terms of the difficulty of the task both groups completed between word learning and the memory test (i.e., count the number of objects in the kitchen, and then replace objects at their previous location). It has been claimed that demanding activities can impair consolidation of memories (Dewar et al., 2007; Wixted, 2004). Indeed, our task was likely to be easier the second time it was performed (i.e., in the Control group), which might have resulted in less impairment of consolidation than in the Novelty group, counter-acting any advantage of novelty. While this is possible, we note that in most situations, including in real-life, novelty is generally associated with greater cognitive demands (to process the novelty), so this potential confound would appear to apply previous demonstrations too, such as novel lessons in children’s schooling (Ballarini et al., 2013; Ramirez Butavand et al., 2020).

In general, evidence for behavioural tagging in humans is still scarce, with several other recent null findings (Biel & Bunzeck, 2019; Biel et al., 2020). Nonetheless, there is also recent study that did find evidence for behavioural tagging in high-school students (e.g., Ramirez Butavand et al., 2020), plus a further study that used familiar and novel Minecraft environments and found a retroactive effect, but only for attention deficit hyperactivity disorder patients and not for typically developing children/adolescents (Baumann et al., 2020). Another study failed to find an effect of surprising actions within video clips on memory for other actions that happened before (Ben-Yakov et al., 2021); though surprise and novelty might function somewhat differently in their effects on memory (Quent et al., 2021). While the evidence in animal studies is more consistent, it is worth noting that the proactive effect of VR on human memory reported by Schomaker and colleagues (Schomaker et al., 2014; Schomaker & Wittmann, 2021) is unlikely to represent the behavioural tagging-like processes seen in animal experiments, because the latter is assumed to take time to influence memory consolidation, whereas Schomaker and colleagues tested memory immediately after the VR experience.

Despite the heterogeneous state of the literature on retroactive/proactive effects of novelty on memory, the idea of behavioural tagging remains strong and extends to manipulations other than novelty, such as manipulations of post-encoding stress (Lopes da Cunha et al., 2018; Quent et al., 2018; Ritchey et al., 2017), fear conditioning (Dunsmoor et al., 2015; Hennings et al., 2021), physical exercise (Roig et al., 2013, 2016), post-encoding arousal (e.g., Nielson & Arentsen, 2012), and reward (e.g., Patil et al., 2017). While there are several indirect demonstrations that (behavioural) tagging affects weakly encoded information (Baumann et al., 2020; Lopes da Cunha et al., 2018; Quent et al., 2018), further systematic work is needed to provide direct evidence for the secondary hypotheses tested in here, i.e., that tagging should benefit weak memories more than strong ones, and differentially affect the subsequent experience of recollection versus familiarity.

In summary, one fruitful avenue for future work would be to manipulate novelty in ways that are not confounded by cognitive demand. However, the behavioural tagging hypotheses can also be tested by using other manipulations like stress, arousal, and physical exercise, which may be easier to de-confound from cognitive demand, and combined with the weak/strong encoding task that we used here. It is also important to register such future experiments (as here), just in case the positive effects in the literature are false positives, and many other negative results are simply not reported (and to get a more accurate indication of the size of any effect, e.g., for meta-analyses).
